# Identification of Key Candidate Genes Involved in the Progression of Idiopathic Pulmonary Fibrosis

**DOI:** 10.3390/molecules26041123

**Published:** 2021-02-20

**Authors:** Yu Cui, Jie Ji, Jiwei Hou, Yi Tan, Xiaodong Han

**Affiliations:** 1 Immunology and Reproduction Biology Laboratory & State Key Laboratory of Analytical Chemistry for Life Science, Medical School, Nanjing University, Hankou Road 22, Nanjing 210093, China; cy99416@163.com (Y.C.); jieji1225@163.com (J.J.); houjiwei3707@126.com (J.H.); TANYIZHIYOU18sui@163.com (Y.T.); 2 Jiangsu Key Laboratory of Molecular Medicine, Nanjing University, Nanjing 210093, China

**Keywords:** IPF, MMP, OPN, IGF-1, intergrin

## Abstract

Idiopathic pulmonary fibrosis (IPF) is a lethal, agnogenic interstitial lung disease with limited therapeutic options. To investigate vital genes involved in the development of IPF, we integrated and compared four expression profiles (GSE110147, GSE53845, GSE24206, and GSE10667), including 87 IPF samples and 40 normal samples. By reanalyzing these datasets, we managed to identify 62 upregulated genes and 20 downregulated genes in IPF samples compared with normal samples. Differentially expressed genes (DEGs) were analyzed by gene ontology and Kyoto Encyclopedia of Genes and Genomes (KEGG) pathway analysis to illustrate relevant pathways of IPF, biological processes, molecular function, and cell components. The DEGs were then subjected to protein–protein interaction (PPI) for network analysis, serving to find 11 key candidate genes (ANXA3, STX11, THBS2, MMP1, MMP9, MMP7, MMP10, SPP1, COL1A1, ITGB8, IGF1). The result of RT-qPCR and immunohistochemical staining verified our finding as well. In summary, we identified 11 key candidate genes related to the process of IPF, which may contribute to novel treatments of IPF.

## 1. Introduction

Idiopathic pulmonary fibrosis (IPF) is a chronic, devastating, progressive, and fibrotic interstitial lung disease of unknown etiology with few treatment options. IPF occurs primarily in middle-aged and elderly adults, and its median survival time varies from three to five years after diagnosis [1]. The main clinical features of IPF are inexorable decline in lung function, progressive respiratory failure, and high mortality. A nonproductive cough and progressive exertional dyspnea are key symptoms of IPF. The important physical findings are scalene muscle hypertrophy, bibasilar fine crackles, and finger clubbing. Serial changes of forced vital capacity (FVC) are effective predictors of mortality in IPF. Recent international guidelines have emphasized the importance of chest high-resolution computed tomography (HRCT) findings such as subpleural distribution, lower lung field honeycombing, traction bronchiectasis (TBE), and subpleural reticular opacity [2]. The histopathological hallmarks of the disease are alveolar structure collapse, fibroblastic foci formation, and excessive deposition of extracellular matrix (ECM). MMPs are a family of proteinases whose active sites contain zinc, playing a major role in the degradation and remodeling of the extracellular matrix (ECM). The mRNA levels of MMP1, MMP7, MMP9, and MMP10 are significantly upregulated in lung tissues from IPF patients compared with normal lung tissues [3]. The upregulation of MMP1 is associated with the accumulation of both type I and III collagen in IPF. MMP7 levels relate to the severity of lung function impairment, and MMP7 has been evaluated as a biomarker for IPF. Elevations of MMP9 are involved in the alveolar damage and are a marker of activated neutrophils. However, the study of MMP10 in the pathogenesis of IPF is still lacking. IGF1 plays a crucial role in inducing cell proliferation, promoting lung fibroblasts migration, and stimulating collagen synthesis; it is considered an important biomarker in IPF lung tissues [4]. Integrins are cell-surface adhesion molecules that mediate cell–cell, cell–extracellular matrix, and cell–pathogen interactions, and integrin αvβ8 can promote pulmonary fibrosis through the unique TGF-β activation mechanisms [5]. OPN (SPP1) is mainly overexpressed by alveolar epithelial cells and alveolar macrophages in IPF lung tissues; it can induce a fibrotic environment by promoting the increase of type I collagen in fibroblasts [6]. Antifibrotic medications such as pirfenidone and nintedanib have been developed to improve patient quality-of-life [7]. However, the treatment response to these drugs varies, and they only slow the progression of IPF; therapies that can reverse disease progression are still lacking because of an incomplete understanding of the pathology. Therefore, figuring out critical genes involved in the development of IPF is urgent and plays an indispensable role in making progress in the treatment plan. In this study, we aimed to use a bioinformatics approach to uncover candidate genes that are involved in the pathogenesis of IPF and to provide promising biomarkers for the diagnosis and treatment of patients with IPF. Moreover, identification of the common DEGs, their function, and the pathway analysis may contribute to the comprehension of the molecular mechanism of IPF.

To break the limits of inadequate patient samples and obtain an overall pattern in the development of IPF, we sought four different datasets from the Gene Expression Omnibus (GEO, http://www.ncbi.nlm.nih.gov/geo/ (accessed on 2 December 2020)). The GEO database, a repository that contains adequate independent studies, was a place where we could find datasets containing the results of gene expression profiles of clinical samples. During the research, we downloaded four original datasets, GSE110147, GSE53845, GSE24206, and GSE10667, which involved 87 IPF lung samples and 40 normal lung tissue samples. Despite that these datasets are independent, we used bioinformatics methods to integrate the data, combined with gene expression analysis techniques. Thus, we identified the common DEGs of the four different datasets, which are representative to the development of IPF to some extent. Furthermore, gene ontology (GO) and pathway enrichment analysis were performed, contributing to insights into the functional aspects of relevant genes. The common DEGs could be divided into upregulated genes and downregulated genes. Meanwhile, the common DEGs were collected to construct a protein–protein interaction network (PPI), serving to provide a network among these genes according to their interaction. The analysis showed that there are 11 core genes participating in the pathological changes of IPF, in which ANXA3 and STX11 are downregulated genes while THBS2, MMP1, MMP9, MMP7, MMP10, SPP1, COL1A1, ITGB8, and IGF1 are upregulated genes. To confirm the results, we conducted RT-qPCR and immunohistochemistry experiments on normal and IPF clinical samples, accordingly.

## 2. Results

### 2.1. Identification of the Common DEGs

**Figure 1:**
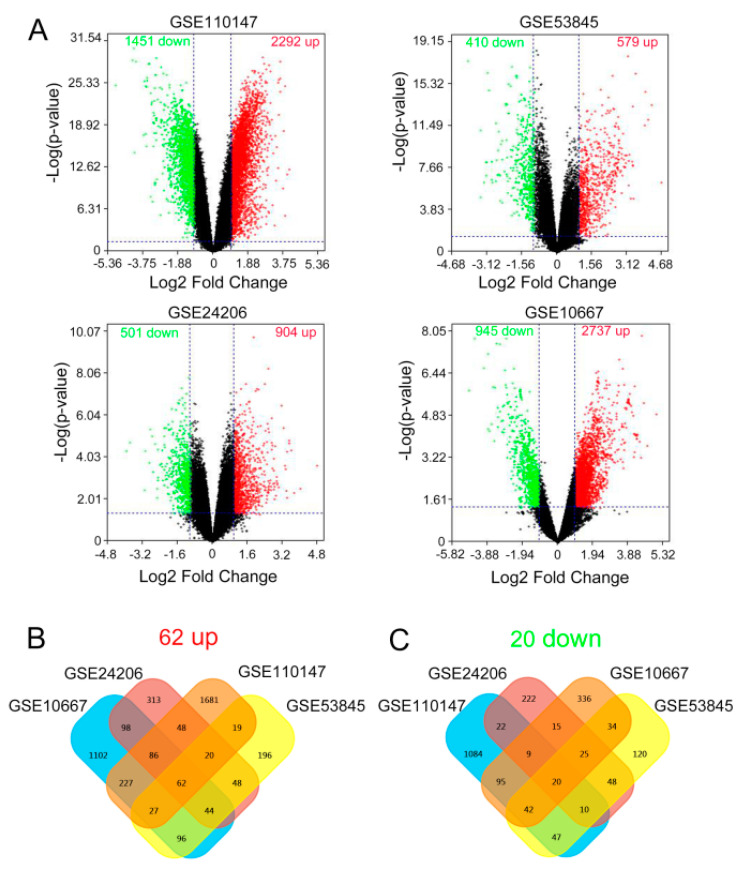
The results of differentially expressed genes (DEGs) in four cohort profile data sets (GSE110147, GSE53845, GSE24206, and GSE10667). (**A**) Volcano plot visualizing the DEGs. The *x* axis indicates the fold change (log-scaled), whereas the *y* axis shows the *p*-values (log-scaled). Vertical lines represent either upregulation or downregulation by 2-fold. The horizontal line represents a *p*-value = 0.05. Red points represent upregulated (up) genes, black points stand for no-significantly-changed (NSC) genes, and green points represent downregulated (down) genes. (**B**) Venn diagrams of the DEGs. Cross areas indicate the commonly upregulated DEGs, and (**C**) cross areas indicate the commonly downregulated DEGs.

Essential information about the samples of the four given datasets is displayed in Table 1. After reanalyzing the four gene expression profiles, we figured out 2292 upregulated genes and 1451 downregulated genes in GSE110147, and there were significant differences in the expression of these genes. Meanwhile, there were 597 upregulated genes and 410 downregulated genes in GSE53845. GSE24206 showed 904 upregulated genes and 501 downregulated genes, while 2737 upregulated genes and 945 downregulated genes could be found in GSE10667. Volcano plots serve to visualize the result accordingly (Figure 1A). Based on this result, we used bioinformatics tools to obtain the common DEGs among GSE110147, GSE53845, GSE24206, and GSE10667. The result shows that there are 62 upregulated genes while there are 20 downregulated genes, accordingly (Figure 1B). Therefore, the common DEGs among the four datasets, including 87 IPF samples and 40 normal samples, were uncovered.

### 2.2. Gene Ontology and Pathway Enrichment Analysis

**Figure 2:**
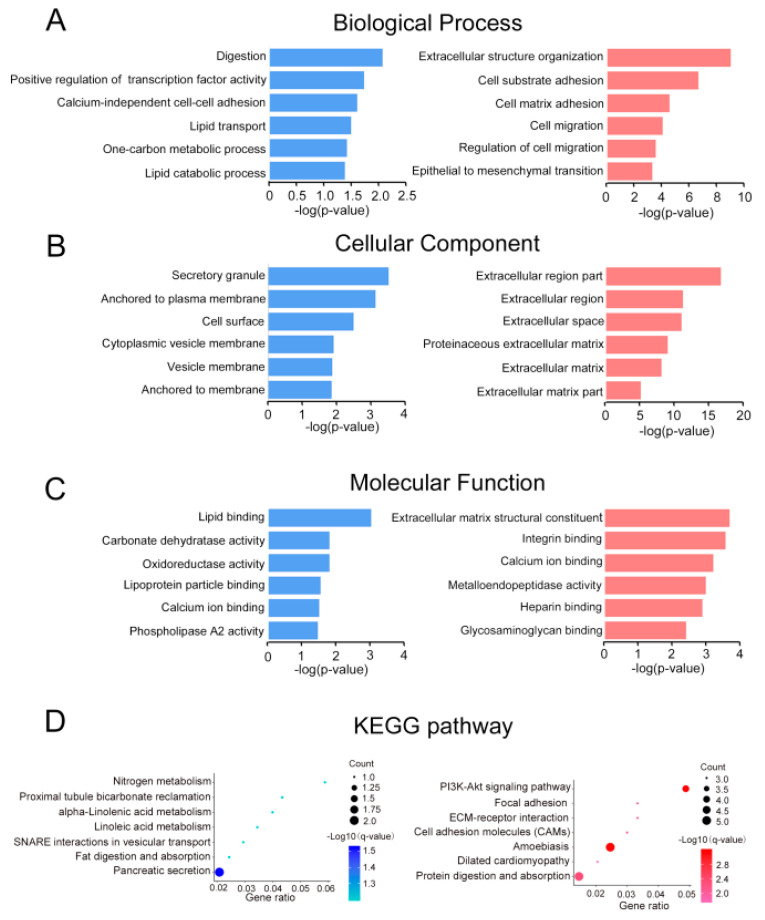
(**A**–**D**) Gene ontology analyses of the common down- (blue) and upregulated (red) genes according to their (**A**) biological process; (**B**) cellular component; (**C**) molecular function; and (**D**) Kyoto Encyclopedia of Genes and Genomes (KEGG) pathway.

The 82 common DEGs were then further analyzed to figure out their functional enrichment, 60 of which had three aspects. For biological process, it demonstrates that downregulated genes mainly participate in digestion, positive regulation of transcription factor activity, calcium-independent cell–cell adhesion, and lipid transport, while upregulated genes enrich in extracellular structure organization, cell–substrate adhesion, cell–matrix adhesion, and cell migration (Figure 2A). When it comes to cellular components, downregulated genes are most related with secretory granule, anchored to the plasma membrane and cell surface, while upregulated genes mainly rich in extracellular region part, extracellular region, and extracellular space (Figure 2B). Moreover, for molecular function, we discovered that downregulated genes enrich in lipid binding, and upregulated genes are concerned with extracellular matrix structural constituent and integrin-binding and calcium-binding (Figure 2C). Apparently, most common DEGs, especially upregulated genes, were significantly enriched in extracellular structure generation. In addition, Kyoto Encyclopedia of Genes and Genomes (KEGG) pathway analysis shows enrichment in pancreatic secretion for downregulated genes and enrichment 72 in protein digestion and PI3k–Akt signaling pathway for upregulated genes.

### 2.3. Establish Protein–Protein International (PPI) Network of the Common DEGs

**Figure 3:**
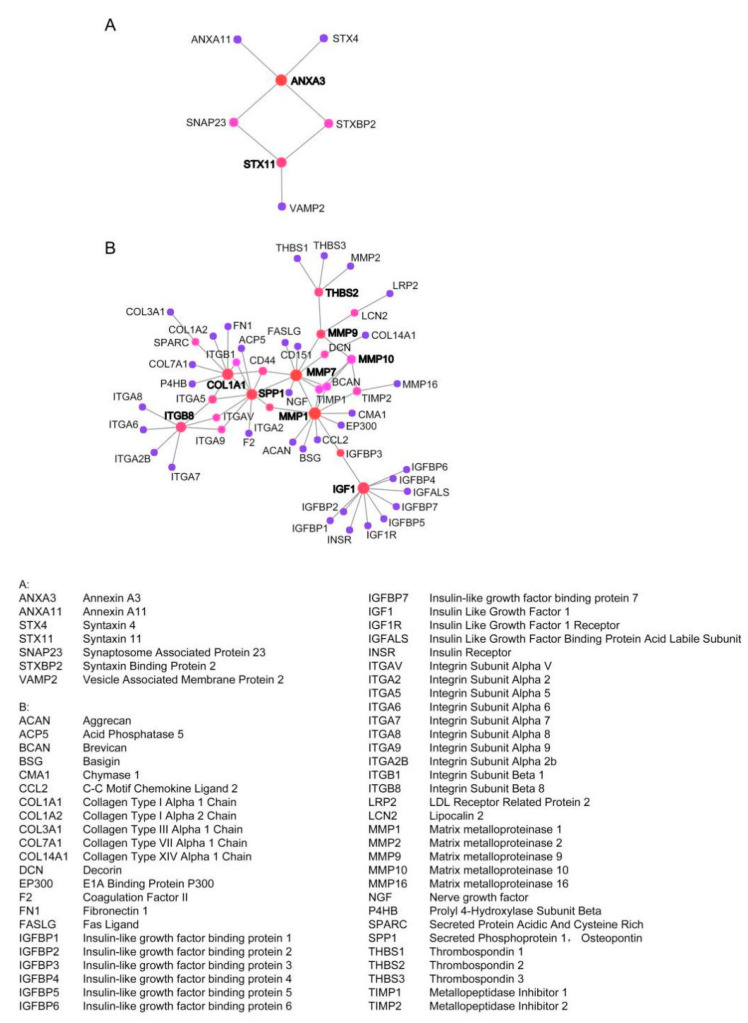
Protein–protein interaction (PPI) network complex and modular analysis of DEGs. (**A**) The PPI network of downregulated DEGs contains 7 nodes (genes) and 7 edges (gene–gene associations). (**B**) The PPI network of upregulated DEGs contains 56 nodes(genes) and 67 edges (gene–gene associations). Associations are meant to be specific and meaningful; genes jointly contribute to a shared function; this does not necessarily mean they are physically binding each other.

PPI network and module analysis PPI network of the DEGs was constructed by the Search Tool for the Retrieval of Interacting Genes (STRING). The interaction relationships among the common DEGs were visualized by using Cytoscape software. A total of 82 common DEGs (62 upregulated genes and 20 downregulated genes) were filtered into the PPI network complex. Each node represents a gene, and the edges stand for the interactions between nodes (the disconnected nodes are not shown); the final networks are shown in Figure 3. The top 11 hub genes were as follows: ANXA3, STX11, THBS2, MMP1, MMP9, MMP7, MMP10, SPP1, COL1A1, ITGB8, IGF1.

### 2.4. Validation of the 11 Key DEGs

**Figure 4:**
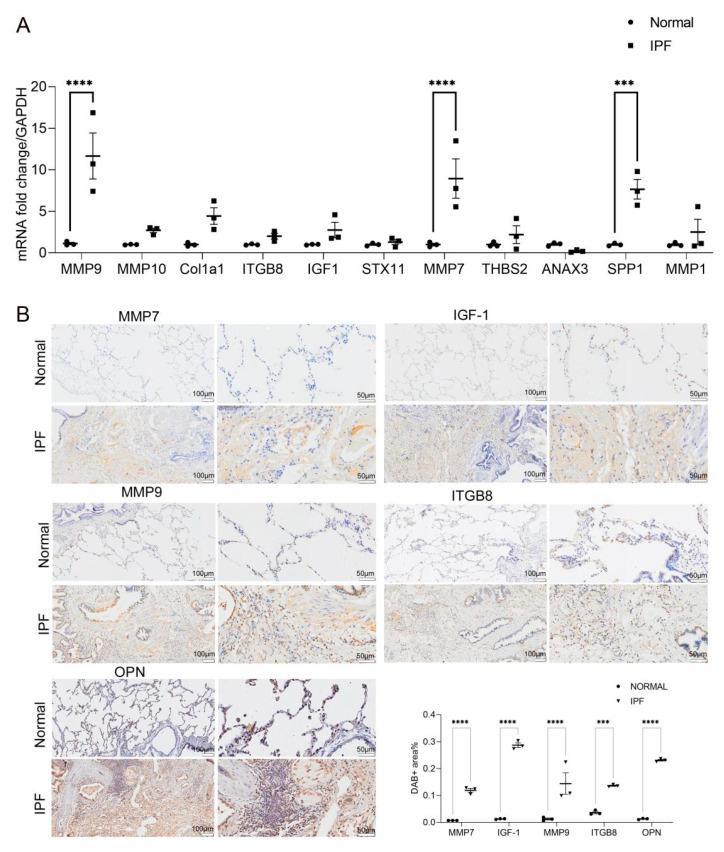
(**A**) Quantitative PCR was used to evaluate the relative expression of candidate genes (ANXA3, STX11, THBS2, MMP1, MMP9, MMP7, MMP10, SPP1, COL1A1, ITGB8, IGF1) in human normal lung tissues (*n* = 3) and IPF lung tissues (*n* = 3), and the relative quantification of the expression of the candidate genes was measured using glyseraldehyde-3-phosphate dehydrogenase (GAPDH) mRNA as an internal control. Data are presented as mean ± SEM (*n* = 3; *** *p* < 0.001, **** *p* < 0.0001; by two-way ANOVA with Duncan’s post hoc test). (**B**) The expression of MMP7, IGF1, SPP1, MMP9, and ITGB8 in human normal lung tissues (*n* = 3) and IPF lung tissues (*n* = 3) was determined by immunohistochemistry (scale bars: left panels = 100 μm, right panels = 50 μm). The DAB Substrate System (DAKO) was used to reveal the immunohistochemical staining; the positive areas in each image were analyzed by Image J software; the percentages of MMP7, IGF1, SPP1, MMP9, and ITGB8 areas were quantified (*** *p* < 0.001, **** *p* < 0.0001).

Finally, we identified 11 candidate genes according to the significant changes in expression levels among the common DEGs and the degree of connectivity within the PPI network. To validate the result, 11 key DEGs (ANXA3, STX11, THBS2, MMP1, MMP9, MMP7, MMP10, SPP1, COL1A1, ITGB8, IGF1) were chosen to test in clinical samples. The result of RT-qPCR (Figure 4A) shows that MMP9, MMP7, and SPP1 were upregulated in the lung tissue of IPF patients predominantly. Moreover, combined with immunohistochemistry staining (Figure 4B), the semi-quantitative analysis of immunohistochemistry by using Image J (https://imagej.nih.gov/ij/ (accessed on 2 December 2020)) showed that IGF1 and ITGB8 were also upregulated in the lung tissues of IPF patients. Additionally, ANXA3 is downregulated in the lung tissues of IPF patients.

## 3. Discussion

The mechanisms underlying the pathogenesis of IPF are quite complex and remain incompletely understood. Increased proliferation, migration, and activation of fibroblasts, as well as inflammation and oxidative stress are all involved in the etiology of IPF. Excessive production of extracellular matrix components also contributes to pulmonary fibrosis. The currently available treatments for IPF are limited; however, the incidence of this disease is rising globally, with associated high morbidity, mortality, and growing economic healthcare burden. Therefore, there is a drive to identify novel biomarkers to find potential therapeutic targets for IPF. We integrated four expression profiles, including 87 IPF samples and 40 normal samples and successfully found 11 key candidate genes (ANXA3, STX11, THBS2, MMP1, MMP9, MMP7, MMP10, SPP1, COL1A1, ITGB8, IGF1) related to the pathogenesis of IPF.

### 3.1. MMP

Matrix metalloproteinases (MMPs) are proteases that degrade all extracellular matrix and many non-matrix protein components. It was initially thought that MMP could limit pulmonary fibrosis by degrading ECM proteins. Nevertheless, recent studies reported that MMPs are involved in the regulation of protein activity other than ECM proteins, including latent growth factors, inflammatory mediators, cleavage of cell surface molecules, antifibrotic growth factors, and receptors [8]. Additionally, in most MMP-deficient mouse pulmonary fibrosis (PF) model research, MMPs promote pulmonary fibrosis responses to injury. MMP expression is strictly regulated to exert its biological function. Under various pathological conditions (such as asthma and PF), MMPs can induce cell activation, proliferation, apoptosis, and migration [9].

Based on the clinical report that the levels of MMP1, MMP7, MMP8, and MMP9 in IPF blood and/or lung samples are elevated, previous studies proposed the role of MMPs in IPF mechanisms. Those include: (1) promote the transition from epithelium to mesenchyme (MMP3 and MMP7); (2) increase the activity or lung levels of the profibrotic mediators or decrease lung levels of antifibrotic mediators (MMP3, MMP7, and MMP8); (3) promote aberrant migration of epithelial cells and other processes of abnormal repair (MMP3 and MMP9); (4) induce lung macrophage phenotypes to switch from M1 to M2 types (MMP10 and MMP28); and (5) promote migration of fibrocyte (MMP8) [10].

In our study, the upregulated expression of MMP1, MMP7, MMP9 was consistent with previous research. Moreover, as a complement to other studies, our finding suggests that MMP10 may also serve as an IPF biomarker.

#### 3.1.1. MMP7

MMP7 is expressed by mononuclear phagocytes, pulmonary epithelial cells, and fibrocytes [11], and it is one of the most elevated genes in fibrotic lungs. MMP7 has extensive substrate affinity for extracellular matrix components, including type IV collagen, laminin, elastin, fibronectin, gelatin, and osteopontin (a multifunctional cytokine regulating cellular migration and adhesion) [12]. MMP7 is also able to process a variety of bioactive substrates and activate proteases, including itself and pro-MMP1, pro-MMP2, and pro-MMP9. MMP7 is a major regulator of TGF biological activity, which can promote collagen synthesis and fibroblasts proliferation. Therefore, the function of MMP7 may be pleiotropic in pulmonary fibrosis, attributed to its various biological functions related to apoptosis, inflammation, fibroproliferation, and innate immunity [13]. By analyzing the gene expression of human fibrotic lung, MMP7 was found to act as a profibrotic mediator in the development of PF. MMP7 null mice are relatively protected from bleomycin-induced fibrosis, suggesting that MMP7 is a central driver of fibrotic tissue responses. According to reports, plasma and broncho-alveolar lavage fluid (BALF) MMP7 levels in patients with IPF are elevated, and the level of MMP7 in plasma has been confirmed as a biomarker of IPF [14]. Meanwhile, in IPF lungs, MMP7 is expressed by macrophages and airway epithelial cells. MMP7 works as sheddase for syndecan-1 in damaged lung epithelial cells. It can carry the CXCL1 as cargo on the glycosaminoglycan chains, and thus releasing the syndecan-1-CXCL1 complexes that are indispensable for neutrophil transepithelial infiltration [15]. This MMP7-dependent neutrophil infiltration promotes fibrosis by facilitating damage of epithelial cells.

#### 3.1.2. MMP9

MMP9 is predominantly expressed by all fibroblasts, epithelial cells, leukocytes, and endothelial cells. Without being produced by resident cells in healthy lungs, MMP9 can be produced by bronchial epithelial cells, Clara cells, alveolar type II cells, fibroblasts, smooth muscle cells, and endothelial cells under various forms of stimulation [16]. Further, MMP9 gene expression was found raised with elevated protein in IPF and experimental lung fibrosis [17]. Compared with normal cells, fibroblasts and alveolar macrophages extracted from the lungs of IPF patients produced a higher level of MMP9. Besides, MMP9 promotes fibrosis by converting transforming growth factor-β (TGF-β) from an inactive latent form to an active form, which stimulates the release of TGF-β homodimers from the latency-related peptide (LAP) and latent TGF-β binding protein-1 (LTBP1).

### 3.2. IGF1

Insulin-like growth factors (IGF1 and IGF2) are insulin-related polypeptides that are significant for regulating cell proliferation and inhibiting programmed cell death [18,19]. These factors are synthesized in almost all tissues of the human body and mainly mediate endocrine, paracrine, and autocrine effects through their type 1 receptor (IGF1R) [20]. It has been demonstrated that IGF1 directly stimulates collagen synthesis and fibroblast proliferation [21]. IGF1 is an influential survival factor involved in the homeostasis and development of various organs and cells by inhibiting cell apoptosis and inducing cellular proliferation [18]. IGF1 was initially identified as an alveolar macrophage-derived growth factor in the lungs [22]. The upregulated expression of IGF1 was validated by immunohistochemistry staining results in our study, and previous findings showed that IGF1 level increased in the bronchoalveolar lavage fluid of patients with IPF and the murine pulmonary fibrosis model, suggesting that IGF1 is involved in the pathogenesis of disease where fibrosis is the predominant feature. Studies showed IGF1 is upregulated in epithelial cells and macrophages in IPF patients. IGF1 mRNA levels also increased in bleomycin-induced pulmonary fibrosis in mice and human IPF [23], indicating that IGF1 has a certain effect on the local production in the fibrosis disease progression.

Extracellular matrix synthesis and collagen deposition are the hallmarks of pulmonary fibrosis. Elevated collagen deposition has been verified to be an indicator of unsatisfied prognosis for patients who have fibrotic lung disorders. It has been confirmed that during the fibro-proliferative stage, the increased expression of collagen III in the lung is related to the development of fibrosis, while the production of collagen I is related to the remodeling of fibrous tissue. Studies have reported a significant correlation analysis of the relationship between IGF1 reactivity and collagen I and collagen III in lung biopsy specimens [24]. Cellular proliferation is another hallmark of fibroproliferation, and IGF1 promotes cell cycle progression. Previous studies showed a significant positive correlation between IGF1 and proliferating cell nuclear antigen (PCNA) immune activity (*p* < 0.001), suggesting that IGF1 is likely to play an extensive role in inducing cell proliferation instead of rigidly promoting fibroblasts proliferation or differentiation. IGF1 induced lung fibroblasts migration in mice. In pulmonary fibrosis, it can be observed that fibroblasts migrate to the intra-alveolar space with extracellular matrix protein deposition. Through the transwell filter system, it was found that IGF1 promoted the migration of lung fibroblasts in mice compared with serum-containing medium. Furthermore, blocking antibody treatment of IGF1R (A12) can abrogate the increased migration [25]. The expression of α-SMA is another marker of pulmonary fibrosis, and blocking the IGF1 pathway in vivo has been reported to reduce α-SMA expression after injury. In mouse injury models, IGF1 receptor blockade accelerated the resolution of fibrosis [26].

### 3.3. Integrin

Integrins are a large family of heterodimeric cell–surface adhesion transmembrane receptors, which mediate cell–extracellular matrix, cell–cell, and cell–pathogen interactions. In mammals, specific and noncovalent bindings of 18 α-subunits to 8 β-subunits were contributed to form 24 diverse integrins. When integrins bind to extracellular ligands, they will activate a series of signaling pathways related to regulating cell cycles, moving receptors to cell membranes and organizing intracellular cytoskeleton. Almost all integrins are linked to the actin cytoskeleton via kindlin-binding motifs and talin in their β-subunit cytoplasmic domains, promoting cell migration, except integrin αvβ8 (differentially expressed in lung adenocarcinoma (DAL-1) through its β8 subunit binding), and integrin α6β4 (linked to the intermediate filament through its β4-subunit). Among the 24 integrins, 8 (including all five integrin-containing αv: αvβ1, αvβ3, αvβ5, αvβ6, and αvβ8) bind ligands via the arginine−glycine−aspartic acid (RGD) sequence [27].

TGF-β is the central cytokine that mediates the homeostasis of mesenchymal, epithelial, and immune cells. Excessive TGF-β signaling by fibroblasts has been shown in numerous fibrotic conditions in the lung, such as pulmonary and airway fibrosis, by regulating the profibrogenic critical components of fibrosis [28]. Integrin-mediated TGF-β activation is crucial to regulate tissue fibrosis and acute lung injury. TGF-β is released from the cell noncovalently, which is related to its pro-peptide (also known as the latency associated peptide (LAP)), producing a latent TGF-β complex that is sequestered in the extracellular matrix (ECM) by binding to ECM glycoproteins [28].

Integrin αvβ8 is expressed by epithelial cells and fibroblasts in the lung—the immunohistochemistry staining results in our study also confirmed this—while αvβ6 is expressed exclusively by epithelial cells. Patients with fibrosis have overexpressed αvβ6 receptors in the lung tissue, and inhibit receptors with a monoclonal antibody can prevent pulmonary fibrosis in mammals. Both αvβ6 and αvβ8 are important in stimulating latent complexes of the growth factor TGF-β. Several integrins can bind to the latent TGF-β complex via RGD binding motifs in the extracellular domains. This interaction enables integrins to function as a direct link between cells and the latent TGF-β complex in the ECM and allows integrins to activate the latent TGF-β complex. Most TGF-β activating integrins (αvβ6, αvβ3, αvβ5, and αvβ1) activated TGF-β via mechanical transduction of intracellular forces to the latent TGF-β complex [29]. However, αvβ8 integrins have unique TGF-β activation mechanisms, including recruitment of MMP14 and proteolytic cleavage of latent complexes to release active TGF-β molecules [30]. In 2014, Minagawa and colleagues designed an antibody against the αvβ8 integrin, and the test verified that it blocks αvβ8-mediated TGF-β activation with high specificity and low off-target effects [31]; αvβ8 integrins are in a high affinity state that binds to latent TGF-β. They also showed that inhibiting this integrin can protect mice from pulmonary fibrosis. Targeting integrin-mediated TGF-β activation locally in disordered tissues represents a promising approach for the treatment of TGF-β-mediated organ fibrosis and tissue remodeling. Making a decrease in the affinity of integrins to interact with latent TGF-β can sufficiently reduce the activation of TGF-β proteins; thereby, in the case of incomplete abrogation of TGF-β protein activation or other integrin functions such as cell adhesion, it prevents tissue remodeling and potentially maintains homeostatic functions of αvβ8 integrins. The different specific distribution of integrin expression in cells and tissues, as well as the different activation mechanisms of TGF-β expression in different tissues, provide an opportunity to reduce the activation of TGF-β expression under pathological conditions without affecting the normal homeostasis function of TGF-β expression, thus promoting the occurrence of disorders [31].

### 3.4. OPN

Osteopontin (OPN, also known as secreted phosphoprotein 1, SPP1) is a phosphorylated acidic glycoprotein secreted by various cells, including activated T cells, osteoclasts, and activated macrophages [32]. OPN contains an arginine–glycine–aspartate (RGD)-binding motif that can bind to a variety of ligands, including some CD44 isoforms, fibronectin, and the integrin family of adhesion molecules [33,34]. OPN is associated with pathological and physiological processes such as metastasis, malignant transformation, and bone resorption. OPN has also been proven to be a pluripotent cytokine that regulates tissue repair, inflammation, and cellular immune response [35,36].

According to reports, the development of fibrosis is associated with increased expression of OPN in the lungs. OPN increased more than 20-fold in IPF lungs, and it was one of the most upregulated genes among the genes that distinguished IPF lungs [37]. Meanwhile, the elevated level of OPN coincides with the RT-qPCR result in our study. OPN strongly mediates adhesion, proliferation, and migration in vitro. It is localized and overexpressed mainly in alveolar macrophages and alveolar epithelial cells of IPF lungs, leading to a prominent increase in proliferation and migration of fibroblasts and epithelial cells. The proliferation of epithelial cells mainly depends on CD44 and the migration mainly depends on CD44 and integrin signaling, while the migration and proliferation of fibroblasts primarily depend on integrins.

OPN has a profibrotic effect on molecules involved in the remodeling of the extracellular matrix. It promoted the increase of type I collagen expression in fibroblasts and MMP7 expression in alveolar epithelial cells. The increased expression of type I collagen genes indicates that OPN can promote the fibrotic environment through its effects on epithelial cells and induce a nondegradative microenvironment similar to the one observed in experimental pulmonary fibrosis and IPF [38]. OPN and MMP7 have been proposed as positive feedback mechanisms: MMP7 activates and cleaves OPN and OPN induces MMP7 in epithelial cells. The positive feedback mechanisms were also supported by the co-localization of MMP7 and osteopontin in IPF epithelial cells. Further, applying the weakest link model to microarray data indicates that the interaction of these two genes can affect the phenotype of IPF [37]. MMP7 and OPN are the target genes of β-catenin [39,40], and the activation of Wnt/β-catenin in IPF lungs was previously reported [41]. Abnormal activation of the Wnt/β-catenin pathway induces the occurrence of OPN and MMP7 and affects the function and expression of the other genes, respectively. In addition, the interaction between MMP7 and OPN in IPF could be related to the deteriorating nature of the disease.

As shown in previous studies, in vivo administration of anti-αv integrin antibodies (RMV-7) that interfere with the binding of OPN and fibroblasts significantly attenuated bleomycin-induced pulmonary fibrosis. Moreover, RMV-7 almost completely eliminates the influence of OPN on in vitro adhesion, proliferation, and migration of fibroblasts. Those findings strengthened the relationship between the OPN–αv integrin interaction and the pathogenesis of pulmonary fibrosis [42].

The proliferation and migration of fibroblasts and the accumulation of extracellular matrix are important in the pathogenesis of pulmonary fibrosis, and the interaction of OPN with collagen, fibronectin, and proteoglycan has been confirmed [43]. Studies showed that OPN directly binds to collagen type I, II, III, IV, and V, which can form a stable complex with fibronectin, indicating that OPN also plays an important role in the synthesis and/or turnover of matrix components.

The high-quality study of Xu et al. has updated and enriched our knowledge about the genes involved in the molecular mechanism of IPF [44]. They screened out 10 core genes (VCAM1, CDH1, CXCL12, JUN, CTGF, SERPINE1, CXCL1, EDN1, COL1A2, and SPARC) through bioinformatics analysis from the datasets GSE2052 and GSE35145, providing a theoretical basis for exploring the novel therapeutic targets for IPF diagnosis and treatment. Both COL1A1 and SPARC genes associated with the disease in Xu’s study coincide with our study results, and they provide general information on the two genes that improved our understanding. In addition, as an improvement of our study, the validation of the candidate genes with RT-qPCR and immunohistochemistry staining enhanced the credibility of the results.

Compared with other similar studies, the genes screened by integrating different databases in our study are more representative. More importantly, we validated the candidate genes using RT-qPCR and immunohistochemistry staining to make the results more credible. Although some of the candidate genes have been explained in previous studies, their clinical applications are still lacking. Our functional enrichment analysis and PPI network indicate that combining multiple markers rather than a single protein intervention may be more helpful for clinical diagnosis and treatment. SPP1, THBS2, ITGB8, and some other genes screened out in this study have rarely been studied in the pathogenesis of pulmonary fibrosis, which also suggests possible approaches for subsequent pathogenesis studies.

To sum up, this study identified several genes associated with IPF through bioinformatics methods, which may provide valuable new ideas for diagnosis and treatment. However, the accurate and direct connections with IPF are still lacking and further studies are needed.

## 4. Materials and Methods

**Figure 5:**
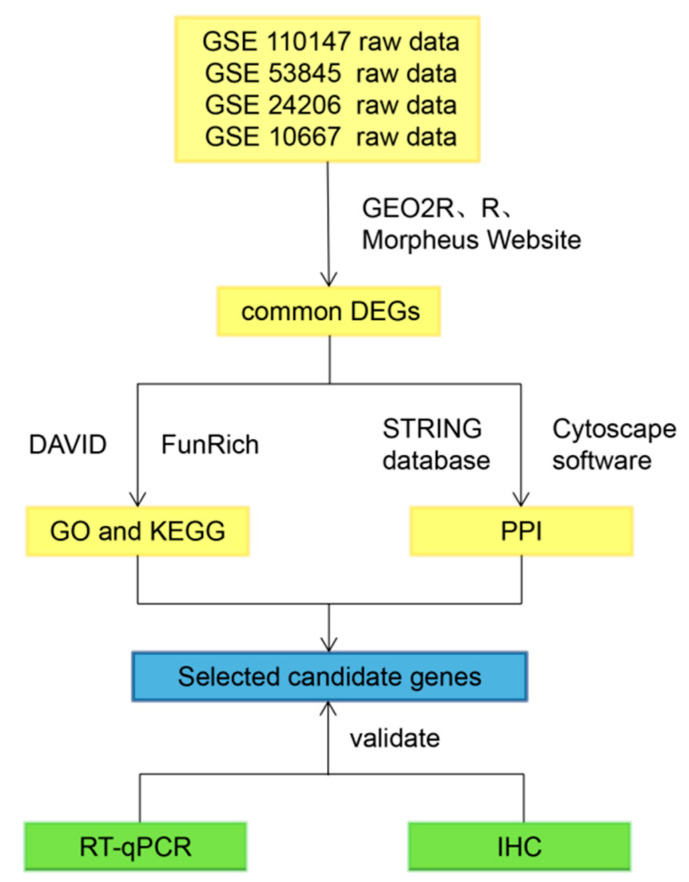
Workflow of this study. GO = gene ontology; PPI = protein–protein interaction network; IHC = immunohistochemistry

Figure 5 shows the workflow of our study.

### 4.1. Data Collection and Analysis

The original gene-expression-profiling datasets were obtained from NCBI GEO databases. On the GEO home page, “IPF” was used as the search term. We selected datasets according to the following criteria: (1) the samples for the study contained lung tissues from healthy donors and patients with IPF; and (2) the datasets provided raw data. Finally, we selected four datasets, GSE110147, GSE53845, GSE24206, and GSE10667. In addition, although GSE10667 and GSE110147 contained samples from patients with usual and nonspecific interstitial pneumonia, respectively, we excluded that information from the analysis, and 17 IPF samples from GSE24206 were derived from only 11 IPF patients (6 patients provided a pair of samples from upper and lower lobes; 5 patients contributed singleton samples.). Four gene expression profiles were analyzed with the online tool GEO2R, the accession numbers of which were GSE110147, GSE53845, GSE24206, and GSE10667, respectively. Among these datasets, GSE110147 was generated from Canada, while the others were generated from the United States. All the profiles could be divided into two groups according to sample types—normal and IPF. The GSE110147 data included 11 normal pulmonary tissues and 22 IPF tissues; the GSE53845 data included 8 normal lung tissues and 40 IPF tissues, and the GSE24206 data included 6 normal lung tissues and 17 IPF tissues. The GSE10667 data included 15 normal pulmonary tissues and 8 IPF tissues. Accordingly, to screen out differentially expressed genes (DEGs) between two sample groups in each gene expression profile, cut-off criteria were set at *p*-value < 0.05 and logFC (>1 or <1). Hierarchical clustering analysis and the Genesis program were employed to generate volcano plots to visualize the results mentioned above. Furthermore, different common expression genes were figured out using the Morpheus Website (available online: https://software.broadinstitute.org (accessed on 2 December 2020)); a Venn diagram was generated to represent the result. The Limma R package was used for DEG screening, while the intersect function in R was employed for identifying the common DEGs.

### 4.2. Gene Ontology and Pathway Enrichment Analysis

To realize the gene ontology enrichment analysis, we input the common DEGs into The Database for Annotation, Visualization and Integrated Discovery (DAVID, https://david.ncifcrf.gov/ (accessed on 2 December 2020)). Meanwhile, the functional enrichment analysis tool (FunRich, version: FunRich 3.0, http://www.funrich.org/ (accessed on 2 December 2020)) was employed to achieve pathway enrichment analysis. A *p*-value < 0.05 was considered as statistically significant, with the GO results ranked by *p*-value.

### 4.3. Identification of Protein–Protein Interaction Network (PPI)

The common DEGs were submitted into the FunRich tool by group, which included 62 upregulated genes and 20 downregulated genes, accordingly. Therefore, we managed to construct the protein–protein interaction (PPI) network and figure out central genes participating in the process of IPF. We input the common DEGs in the STRING database (http://string-db.org/ (accessed on 2 December 2020)) to construct the PPI network, and then we visualized the results using Cytoscape software (https://cytoscape.org (accessed on 2 December 2020), v.3.7.0). the *k*-means clustering, only selecting experimentally validated interactions with a combined score > 0.4, which is regarded as crucial.

### 4.4. Clinical Sample Collection

Surgical biopsy specimens from IPF lung tissues were obtained from Nanjing Drum Tower Hospital. Control subjects, similar in age to IPF patients, were selected from patients with nonfibrotic lung disorders. Lung biopsy samples were processed with standard techniques for immunohistochemistry and real-time quantitative PCR analyses. All protocols concerning the use of patient samples in this study were approved by the Ethics Committee at Nanjing Drum Tower Hospital.

### 4.5. Real-Time Quantitative PCR

Total RNA was isolated using Trizol reagent according to the manufacturer’s instructions. cDNA synthesis was conducted with HiScript Q RT SuperMix (Vazyme, Nanjing, China). The qRT-PCR analysis was carried out using SYBR Green I mix (Takara, Dalian, China). The mRNA expression was measured as previously described [39]. Primer pairs used are listed in Table 2. Fold changes in gene expression were determined using the relative comparison method with normalization to glyseraldehyde-3-phosphate dehydrogenase (GAPDH) as an internal loading control.

### 4.6. Immunohistochemical Staining

The immunohistochemistry staining was performed as described previously [45]. Details of the primary and secondary antibodies are provided in Table 3.

## Figures and Tables

**Table 1:** Details about the datasets used in this study.

Accession	Year	Area	Platform	Number of Samples	
				Normal	IPF
GSE10667	2008	USA	GPL4133	15	8
GSE24206	2010	USA	GPL570	6	17
GSE110147	2018	Canada	GPL6244	11	22
GSE53845	2014	USA	GPL6480	8	40

**Table 2:** Primers used for PCR analysis.

Primer	
Homo mmp1R	GTTGTCCCGATGATCTCCCC
Homo mmp7 F	GTCTCTGGACGGCAGCTATG
Homo mmp7 R	GATAGTCCTGAGCCTGTTCCC
Homo anxa3 F	CCTTCGCTCGCAGTTTGTTC
Homo anxa3 R	TCGGTGTCCAACCCAGATAGA
Homo stx11 F	TGAATCGGGTCATGGAAGGTT
Homo stx11 R	CTTGAGGCTGCTCCATGTCT
Homo thbs2 F	TTGGCAAACCAGGAGCTCAG
Homo thbs2 R	GGTCTTGCGGTTGATGTTGC
Homo mmp9 F	CTTTGAGTCCGGTGGACGAT
Homo mmp9 R	TCGCCAGTACTTCCCATCCT
Homo mmp10 F	GACAGAAGATGCATCAGGCAC
Homo mmp10 R	GGCGAGCTCTGTGAATGAGT
Homo col1a1 F	GGACACAGAGGTTTCAGTGGT
Homo col1a1 R	GCACCATCATTTCCACGAGC
Homo itgb8 F	AAAGTGCTGAGTAGAGGGCG
Homo itgb8 R	TATTGTCTTCTGGCCGGGATG
Homo igf1 F	TGCTCTCAACATCTCCCATCTC
Homo igf1 R	CATGGTGTGCATCTTCACCTTC
Homo Spp1 F	GCAGCTTTACAACAAATACCCAGAT
Homo Spp1 R	TGGACTTACTTGGAAGGGTCTGTG

**Table 3:** Specifications of primary antibodies.

Antibody	Company	Catalog	Dilution
Anti-MMP-7	Proteintech	10374-2-AP	1:100
Anti-MMP-9	Proteintech	10375-2-AP	1:100
Anti-SPP1	Proteintech	11952-1-AP	1:100
Anti-IGF-1 Anti-ITGB8	Santa Cruz Santa Cruz	sc-74116 sc-514150	1:50 1:50

## Data Availability

Data is contained within the article.

## Sample Availability

Samples of the compounds are not available from the authors.

## Abbreviations

IPF	Idiopathic pulmonary fibrosis
PF	Pulmonary fibrosis
DEGs	Differentially expressed genes
IHC	Immunohistochemistry
STRING	Search tool for the retrieval of interacting genes
MMP	Matrix metalloproteinase
IGF	Insulin-like growth factor
IGF1R	Insulin Like Growth Factor 1 Receptor
OPN	Osteopontin
TGF-β	Transforming growth factor-β
ANXA3	Annexin A3
STX11	Syntaxin 11
THBS2	Thrombospondin 2
SPP1	Secreted Phosphoprotein 1
COL1A1	Collagen Type I Alpha 1 Chain
LTBP1	Latent TGF-β binding protein-1
LAP	Latency-related peptide (LAP)
RGD	Arginine−glycine−aspartic acid
BLM	Bleomycin
ECM	Extracellular matrix
PPI	protein–protein interaction network
KEGG	Kyoto Encyclopedia of Genes and Genomes
GO	Gene ontology
GEO	Gene Expression Omnibus
BALF	broncho-alveolar lavage fluid
DAVID	The Database for Annotation, Visualization and Integrated Discovery
GAPDH	glyseraldehyde-3-phosphate dehydrogenase
PCNA	proliferating cell nuclear antigen

## References

[1] King T., Pardo A., Selman M. (2011). Idiopathic pulmonary fibrosis. Lancet.

[2] Kishaba T. (2019). Evaluation and management of Idiopathic Pulmonary Fibrosis. Respir. Investig..

[3] Craig V.J., Zhang L., Hagood J.S. (2015). Matrix metalloproteinases as therapeutic targets for idiopathic pulmonary fibrosis. Am. J. Respir. Cell Mol. Biol..

[4] Romano G. (2003). The complex biology of the receptor for the insulin-like growth factor-1. Drug News Perspect..

[5] Wang J., Dong X., Zhao B. (2017). Atypical interactions of integrin alphaV beta8 with pro-TGF-beta1. Proc. Natl. Acad. Sci. USA.

[6] Berman J.S., Serlin D., Li X., Whitley G., Hayes J., Rishikof D.C., Ricupero D.A., Liaw L., Goetschkes M., O’Regan A.W. (2004). Altered bleomycin-induced lung fibrosis in osteopontin-deficient mice. Am. J. Physiol. Cell. Mol. Physiol..

[7] Barratt S.L., Creamer A., Hayton C., Chaudhuri N. (2018). Idiopathic Pulmonary Fibrosis (IPF): An Overview. J. Clin. Med..

[8] McKeown S., Richter A.G., O’Kane C., McAuley D.F., Thickett D.R. (2009). MMP expression and abnormal lung permeability are important determinants of outcome in IPF. Eur. Respir. J..

[9] Mahalanobish S., Saha S., Dutta S., Sil P.C. (2020). Matrix metalloproteinase: An upcoming therapeutic approach for idiopathic pulmonary fibrosis. Pharmacol. Res..

[10] Hatfield K.J., Reikvam H., Bruserud O. (2010). The crosstalk between the matrix metalloprotease system and the chemokine network in acute myeloid leukemia. Curr. Med. Chem..

[11] García-De-Alba C., Becerril C., Ruiz V., González Y., Reyes S., García-Alvarez J., Selman M., Pardo A. (2010). Expression of Matrix Metalloproteases by Fibrocytes: Possible role in migration and homing. Am. J. Respir. Crit. Care Med..

[12] Dancer R.C.A., Wood A.M., Thickett D.R. (2011). Metalloproteinases in idiopathic pulmonary fibrosis. Eur. Respir. J..

[13] Kurmasheva R.T., Houghton P.J. (2006). IGF-I mediated survival pathways in normal and malignant cells. Biochim. Biophys. Acta (BBA)-Bioenerg..

[14] Kristensen J.H., Larsen L., Dasgupta B., Brodmerkel C., Curran M., Karsdal M., Sand J., Willumsen N., Knox A., Bolton C. (2015). Levels of circulating MMP-7 degraded elastin are elevated in pulmonary disorders. Clin. Biochem..

[15] Li Q., Park P.W., Wilson C.L., Parks W.C. (2002). Matrilysin Shedding of Syndecan-1 Regulates Chemokine Mobilization and Transepithelial Efflux of Neutrophils in Acute Lung Injury. Cell.

[16] Henry M., McMahon K., Mackarel A., Prikk K., Sorsa T., Maisi P., Sepper R., Fitzgerald M., O’Connor C. (2002). Matrix metalloproteinases and tissue inhibitor of metalloproteinase-1 in sarcoidosis and IPF. Eur. Respir. J..

[17] Yu Q., Stamenkovic I. (2000). Cell surface-localized matrix metalloproteinase-9 proteolytically activates TGF-beta and promotes tumor invasion and angiogenesis. Genes Dev..

[18] Pardo A., Selman M., Kaminski N. (2008). Approaching the degradome in idiopathic pulmonary fibrosis☆. Int. J. Biochem. Cell Biol..

[19] Leroith D., Roberts C.T. (2003). The insulin-like growth factor system and cancer. Cancer Lett..

[20] Jones J.I., Clemmons D.R. (1995). Insulin-Like Growth Factors and Their Binding Proteins: Biological Actions. Endocr. Rev..

[21] Goldstein R.H., Poliks C.F., Pilch P., Smith B.D., Fine A. (1989). Stimulation of Collagen Formation by Insulin and Insulin-Like Growth Factor I in Cultures of Human Lung Fibroblasts. Endocrinology.

[22] Rom W.N., Basset P., A Fells G., Nukiwa T., Trapnell B.C., Crysal R.G. (1988). Alveolar macrophages release an insulin-like growth factor I-type molecule. J. Clin. Investig..

[23] Maeda A., Hiyama K., Yamakido H., Ishioka S., Yamakido M. (1996). Increased Expression of Platelet-Derived Growth Factor A and Insulin-Like Growth Factor-I in BAL Cells During the Development of Bleomycin-Induced Pulmonary Fibrosis in Mice. Chest.

[24] Krein P.M., Sabatini P.J.B., Tinmouth W., Green F.H.Y., Winston B.W. (2003). Localization of Insulin-like Growth Factor-I in Lung Tissues of Patients with Fibroproliferative Acute Respiratory Distress Syndrome. Am. J. Respir. Crit. Care Med..

[25] Choi J.-E., Lee S.-S., Sunde D.A., Huizar I., Haugk K.L., Thannickal V.J., Vittal R., Plymate S.R., Schnapp L.M. (2009). Insulin-like Growth Factor-I Receptor Blockade Improves Outcome in Mouse Model of Lung Injury. Am. J. Respir. Crit. Care Med..

[26] Burtrum D., Zhu Z., Lu D., Anderson D.M., Prewett M., Pereira D.S., Bassi R., Abdullah R., Hooper A.T., Koo H. (2003). A fully human monoclonal antibody to the insulin-like growth factor I receptor blocks ligand-dependent signaling and inhibits human tumor growth in vivo. Cancer Res..

[27] Anderson N.A., Campos S., Butler S., Copley R.C.B., Duncan I., Harrison S., Le J., Maghames R., Pastor-Garcia A., Pritchard J.M. (2019). Discovery of an Orally Bioavailable Pan αv Integrin Inhibitor for Idiopathic Pulmonary Fibrosis. J. Med. Chem..

[28] Tatler A.L., Jenkins G. (2012). TGF-β activation and lung fibrosis. Proc. Am. Thorac. Soc..

[29] Shi M., Zhu J., Wang R., Chen X., Mi L., Walz T., Springer T.A. (2011). Latent TGF-β structure and activation. Nature.

[30] Mu D., Cambier S., Fjellbirkeland L., Baron J.L., Munger J.S., Kawakatsu H., Sheppard D., Broaddus V.C., Nishimura S.L. (2002). The integrin αvβ8 mediates epithelial homeostasis through MT1-MMP–dependent activation of TGF-β1. J. Cell Biol..

[31] Minagawa S., Lou J., Seed R.I., Cormier A., Wu S., Cheng Y., Murray L., Tsui P., Connor J., Herbst R. (2014). Selective Targeting of TGF-β Activation to Treat Fibroinflammatory Airway Disease. Sci. Transl. Med..

[32] Denhardt D.T., Guo X. (1993). Osteopontin: A protein with diverse functions. FASEB J..

[33] Reinholt F.P., Hultenby K., Oldberg A., Heinegard D. (1990). Osteopontin—A possible anchor of osteoclasts to bone. Proc. Natl. Acad. Sci. USA.

[34] Weber G.F., Ashkar S., Glimcher M.J., Cantor H. (1996). Receptor-ligand interaction between CD44 and osteopontin (Eta-1). Science.

[35] Denhardt D.T., Noda M., O’Regan A.W., Pavlin D., Berman J.S. (2001). Osteopontin as a means to cope with environmental insults: Regulation of inflammation, tissue remodeling, and cell survival. J. Clin. Investig..

[36] O’Regan A., Berman J.S. (2001). Osteopontin: A key cytokine in cell-mediated and granulomatous inflammation. Int. J. Exp. Pathol..

[37] Pardo A., Gibson K., Cisneros J., Richards T.J., Yang Y., Becerril C., Yousem S., Herrera I., Ruiz V., Selman M. (2005). Up-Regulation and Profibrotic Role of Osteopontin in Human Idiopathic Pulmonary Fibrosis. PLoS Med..

[38] Selman M., Ruiz V., Cabrera S., Segura L., Ramírez R., Barrios R., Pardo A. (2000). TIMP-1, -2, -3, and -4 in idiopathic pulmonary fibrosis. A prevailing nondegradative lung microenvironment?. Am. J. Physiol. Cell. Mol. Physiol..

[39] Brabletz T., Jung A., Dag S., Hlubek F., Kirchner T. (1999). β-Catenin Regulates the Expression of the Matrix Metalloproteinase-7 in Human Colorectal Cancer. Am. J. Pathol..

[40] El-Tanani M., Platt-Higgins A., Rudland P.S., Campbell F.C. (2004). Ets Gene PEA3 Cooperates with β-Catenin-Lef-1 and c-Jun in Regulation of Osteopontin Transcription. J. Biol. Chem..

[41] Chilosi M., Poletti V., Zamò A., Lestani M., Montagna L., Piccoli P., Pedron S., Bertaso M., Scarpa A., Murer B. (2003). Aberrant Wnt/β-Catenin Pathway Activation in Idiopathic Pulmonary Fibrosis. Am. J. Pathol..

[42] Takahashi F., Takahashi K., Okazaki T., Maeda K., Ienaga H., Maeda M., Kon S., Uede T., Fukuchi Y. (2001). Role of Osteopontin in the Pathogenesis of Bleomycin-Induced Pulmonary Fibrosis. Am. J. Respir. Cell Mol. Biol..

[43] Goldstein R.H., Fine A. (1986). Fibrotic Reactions in the Lung: The Activation of the Lung Fibroblast. Exp. Lung Res..

[44] Xu Z., Mo L., Feng X., Huang M., Li L. (2020). Using bioinformatics approach identifies key genes and pathways in idiopathic pulmonary fibrosis. Medicine.

[45] Hou J., Ma T., Cao H., Chen Y., Wang C., Chen X., Xiang Z., Han X. (2018). TNF-α-induced NF-κB activation promotes myofibroblast differentiation of LR-MSCs and exacerbates bleomycin-induced pulmonary fibrosis. J. Cell. Physiol..

